# The routine use of preoperative non-contrast chest computerized tomography and carotid arteries Doppler prior to cardiac surgery

**DOI:** 10.1186/s13019-022-01927-2

**Published:** 2022-07-23

**Authors:** Ron Nates, Mattan Arazi, Liza Grosman-Rimon, Roy Israel, Jacob Gohari, Leonid Sternik, Erez Kachel

**Affiliations:** 1 The Lydia and Carol Kittner, Lea and Benjamin Davidai Division of Cardiovascular Medicine and Surgery, Padeh Poriya Medical Center, Lower Galilee, Tiberias, Israel; 2 Department of Emergency Medicine, University of Texas Health Science Center, Houston, USA; 3 The Ruth and Bruce Rappaport Faculty of Medicine, Technion-Israel Institute of Technology, Haifa, Israel; 4 The Azriely Faculty of Medicine in the Galilee, Bar-Ilan University, Safed, Zefat, Israel; 5 Creedmoor Psychiatric Center, Queens Village, New York, NY USA; 6 Department of Cardiac Surgery, Sheba Medical Centre, Tel Hashomer, Israel

**Keywords:** Preoperative non-contrast chest computerized tomography, Preoperative carotid arteries Doppler, Cardiac surgery, Unexpected findings

## Abstract

**Introduction:**

There is no consensus as to which patients should undergo Non-Contrast Chest Computerized Tomography (NCCCT) and carotid arteries Doppler (CD) prior to cardiac surgery. The objective of this study was to examine whether preoperative imaging modalities provide clinical benefits and a change in surgical strategy.

**Methods:**

We routinely performed NCCCT and CD in all non-urgent cardiac surgery patients. Major NCCCT/CD findings related to cardiovascular findings (aortic calcification/atherosclerosis, carotid artery plaque/stenosis), or other incidental findings (lung kidney, thyroid, adrenal, gastrointestinal sites etc.) were documented. The results were divided into 3 categories: (A) findings requiring both changes in surgical strategy and post-operative evaluation/treatment; (B) findings requiring changes in surgical strategy, but not requiring a specific post-operative evaluation/treatment; (C) findings not requiring changes in surgical strategy but requiring post-operative evaluation/treatment.

**Results:**

In this cohort, 93 (18.6%) out of 500 patients had significant cardiac and extra-cardiac findings on NCCCT and/or CD. Among the 93 patients with significant findings, 33.33% (31 patients, 6.2% of all patients) were in group A, 7.5% (7 patients, 1.4% of all patients) were in group B, and 59.14% (55 patients, 11% of all patients) were in group C. Change in surgical strategies included, for example, switching from planned on-pump Coronary Artery Bypass Graft surgery (CABG) to off-pump CABG and performing additional procedures to the originally planned heart surgery.

**Conclusion:**

Routine preoperative NCCCT and CD evaluation in all non-urgent cardiac surgical patients is an effective measure for uncovering cardiac and extra-cardiac findings prior to surgery.

## Introduction

Preoperative evaluation of the heart, great vessels and chest with imaging modalities such as echocardiography and chest radiography are performed routinely prior to cardiac surgery for selected patients [1–3]. However, as many cardiac surgical patients today are elderly, high risk with significant comorbidities, these imaging modalities may not fully provide an adequate preoperative assessment for surgical risk reduction and patient management [4–6]. Epi-aortic ultrasound has been proven to be effective for detecting calcified plaques in the ascending aorta, however, is limited due to its intraoperative use, operator experience, time consumption [3, 7] and has restricted use to the aorta.

Non-contrast computed chest tomography (NCCCT), typically reserved only for high risk patients, have demonstrated to change operative strategy as well as significantly reduce morbidity and mortality rates. [7] Therefore, the objective of this study was to determine whether routine screening with these preoperative imaging modalities provide clinical benefits and impact surgical decision making in all patients scheduled for cardiac surgery, as well as identify abnormal findings that may require postoperative evaluation and/or treatment.

## Patients and methods

### Patient’s cohort

A retrospective study was conducted using data from patients who underwent cardiac surgery between May 2015 and January 2019 in the Baruch Padeh Poriya Medical Center, located in northern Israel. The study was approved by the Helsinki Ethics Committee of Poriya Medical Center. All non-urgent patients schedule for heart surgery underwent routine NCCCT (Philips iCT 256) and carotid artery Doppler (Hitachi Aloka Arietta 560 with a Linear 7/5 probe) prior to cardiac surgery. Data were collected from chart review and medical electronic record. Patients’ characteristics included medical history and co-morbidities, type of original heart surgery, risk factors and findings on NCCCT and CD. Age, sex, height and BMI were also included in the data collected. In total, 500 consecutive patients were included in the study. NCCCT and CD imaging were all interpreted by a radiologist and an experienced cardiac surgeon. Analysis of data was completed to identify patients with incidental findings prior to surgery. Cases were then reviewed to examine the impact of findings on patient management.

### Statistical analysis

Statistical analysis was performed using SPSS software version 22.0 (IBM, Armonk, NY, USA). Descriptive statistics was presented for baseline data, including patients’ characteristics and co-morbidities as either means and standard deviation, or rate and percentages. The results were divided into 3 categories: (A) findings requiring both changes in surgical strategy and post-operative evaluation/treatment; (B) findings requiring changes in surgical strategy, but not requiring a specific post-operative evaluation/treatment; (C) findings not requiring changes in surgical strategy but requiring post-operative evaluation/treatment.

## Results

**Fig. 1:**
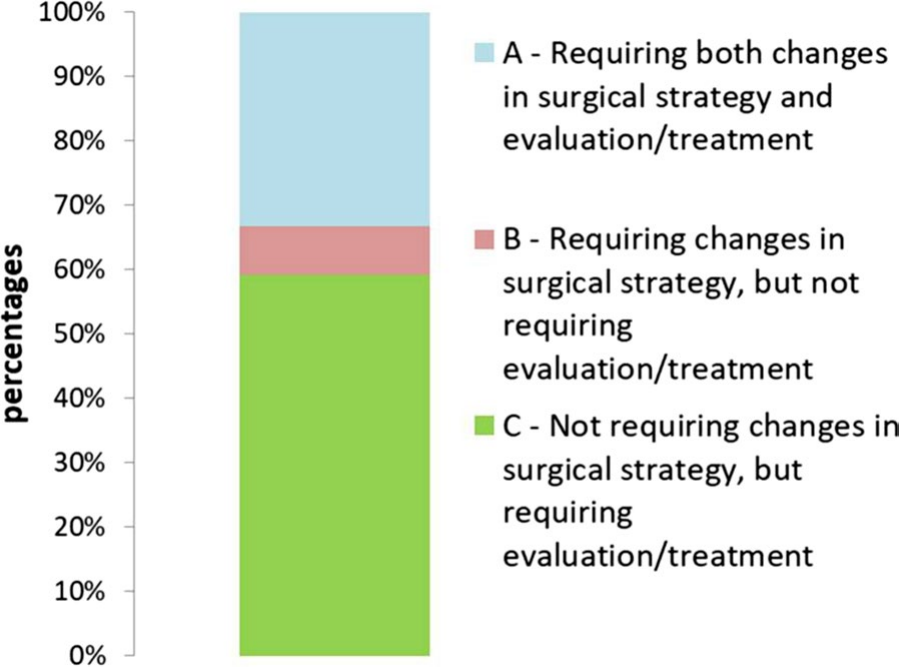
Cardiac and extra-cardiac findings following routine preoperative imaging were divided into 3 categories: **A** findings requiring both changes in surgical strategy and post-operative evaluation/treatment; **B** findings requiring changes in surgical strategy, but not requiring a specific post-operative evaluation/treatment; **C** findings not requiring changes in surgical strategy but requiring post-operative evaluation/treatment

Patients’ characteristics are described in Table 1. In total, 500 charts were reviewed. In our cohort, 64% of the patients underwent CABG, 11% valvular surgery, 21% combined CABG and valvular surgery and 4% other type of cardiac surgery. Among this patient cohort, 93 (18.6%) patients had significant cardiac and extra-cardiac findings on NCCCT and/or CD (Table 2). The mean age of patients was 65.5 years old, with males accounting for 80% of all patients. In our cohort, 66% had hypertension, 67% of patients had hyperlipidemia, 43% of patients had diabetes mellitus (type 1 and 2) and 13.5% of patients demonstrated aortic calcification or atherosclerosis. In Fig. 1 patients were subdivided into various groups—31 patients (33.33%) in group A (6.2% of all patients) required both changes in surgical strategy and evaluation/treatment, 7 patients (7.53%) in group B (1.4% from all cases) required changes in surgical strategy, but did not require a specific post-operative evaluation/treatment and 55 patients (59.14%) in group C (11% of all patients) did not require change in surgical strategy but did require further evaluation/treatment. Main changes in surgical strategy due to NCCCT and CD findings included choosing a more precise site for aortic cannulation and clamping due to severe aortic calcification, shifting from planned on pump Coronary Artery Bypass Graft surgery (CABG) to Off-Pump Coronary Artery Bypass (OPCAB).

**Table 1:** Baseline characteristics of the patient cohort

	Value
Variables	n = 93
Age (yr)	65.5 ± 9.3
Height (cm)	167.9 ± 8.46
Weight (kg)	80.2 ± 13.7
BMI (kg/m^2^)	28.6 ± 4.9
BSA (m^2^)	1.9 ± 0.19
Male Sex (%)	(n = 74) 80%
Diabetes (%)	(n = 40) 43%
Hypertension (%)	(n = 61) 66%
Hyperlipidemia (%)	(n = 62) 67%
Family History of CAD (%)	(n = 8) 9%
Tobacco use (%)	(n = 40) 43%
Aortic calcification/ atherosclerosis (%)	(25) 13.5%

**Table 2:** Number of non-cardiac radiological findings classified by system

Radiological findings by system	n = 93
Pulmonary	39.3% (n = 34)
Carotid	12.7% (n = 11)
Thyroid	11.5% (n = 10)
Adrenal	9.2% (n = 8)
Kidney	13.8% (n = 12)
GI	20.8% (n = 18)

Examples of findings that required further evaluation and/or treatment include adrenal masses, kidney adenomas, and lung pathologies such as ground glass opacities, emphysematous diffuse changes and nodules that required pulmonologist referrals. It is important to note that patients who found to have incidental radiological findings in our department were referred to the appropriate outpatient specialists for further imaging.

In group A, nine patients underwent recannulation and overall, the number of coronary artery bypass grafts was reduced due to significant ascending aorta calcifications. Vascular anastomoses were performed using one or two thoracic arteries and some required direct escalation to proximal anastomoses on the ascending aorta. The rest of the grafts were completed as a hybrid procedure by a post-operative. PCI Seven patients were shifted from a planned full CABG to Off-pump Coronary Artery Bypass (OPCAB) while the rest of the necessary grafts were again, completed as a hybrid procedure via a post-operative PCI. Six patients underwent an additional lobectomy or lung wedge resection to the originally planned cardiothoracic surgery due to pulmonary space occupying lesions (SOL). Three patients were shifted from a planned CABG + valvular surgery to valvular surgery alone and the coronary lesions were treated by PCI. Six patients underwent thymectomy due to thymic lesions and were referred to an oncologist for post-operative follow up. In group B, four patients underwent combined or immediate (two weeks later) carotid endarterectomy due to severe carotid stenosis or an ulcerated carotid plaque. Three patients underwent complete OPCAB surgery instead a planned full bypass CABG due to severe ascending aortic calcifications. In group C, we found 12 renal adenomas, 21 thyroid findings, 9 significant asymptomatic carotid findings, which did not need surgical intervention, 13 kidney or gallbladder stones – all patients with these findings were referred to the appropriate specialists for close follow-up”.

## Discussion

While there are few reports on NCCCT and/or CD performed for patients prior to cardiac surgery in research studies, [3, 4, 7, 8] to the best of our knowledge, our center is the first to introduce this practice as a standard of care for all patients undergoing cardiac surgery. Pre-operative NCCCT and CD may assist in the surgical decision making and selection of optimal strategies to decrease perioperative stroke, morbidity and mortality rates. [9]

Our results showed that almost one in every five patients (18.6% of all patients) undergoing cardiac surgery had a significant finding during preoperative screening. This approach led to a change in their clinical management with 6.2% of all patients requiring both changes in surgical strategy and post-operative evaluation/treatment, 1.4% of all patients requiring changes in surgical strategy alone, and 11% of all patients requiring post-operative evaluation/treatment alone. Overall, we demonstrated that in one of every 13 patients required a change in surgical strategy secondary to preoperative radiological findings.

Commonly, a routine preoperative echocardiogram and chest radiograph are performed, while NCCCT and CD are typically reserved for patients considered high-risk due to age or other risk factors. Otherwise, procedures were completed and unforeseen postoperative complications were attributed to underlying non-modifiable risk factors. In our patient cohort, identified comorbidities such as aortic and carotid calcifications, as well as other extra cardiac findings, led to a modified surgical strategy, which may have improved patient outcomes in the short or long term. [3, 7, 8, 10] One recent study examined preoperative screening for atheromatous aortic disease using computed tomography angiography and found that screening is associated with reduced risk of stroke after coronary artery bypass grafting. [11] Our study included all type of cardiac surgeries and carotid arteries Doppler, allowing more through patient examination as well as included a large spectrum clinical findings.

A meta-analysis by Den Harder et al. [3] demonstrated a 77–96% decrease in perioperative stroke rate and a 49–66% decrease in mortality in 7 studies reviewing preoperative Computerized Tomography (CT) involving primary cardiac surgery. Moreover, Den Harder et al. [3] demonstrated that the use of preoperative chest CT provided excellent visualization of aortic calcifications and assisted in operative planning. Fukuda et al. [8] studied 308 patients who underwent preoperative duplex scanning of the carotid artery, non-contrast CT of the chest, and intraoperative ultrasonography of the ascending aorta. In their study, 29 patients (9.4%) required a change in surgical strategy (i.e., change in cannulation site). Their findings were very similar to ours, with 7.6% of patients requiring changes to surgical approach/strategy.

Lee et al. [7] studied 503 patients of which 114 were identified as high risk for stroke and underwent preoperative CT. Nineteen of the patients receiving a preoperative CT (16.7%) required changes in operative strategy to avoid calcified areas. In contrast to our study, which included all cardiac patients, this study scanned only patients determined to be at high risk, and therefore demonstrated a higher operative strategy change of 16.7% vs. 7.6% in our patient cohort. Interesting to note, Lee et al. [7] also displayed an improvement in mortality and stroke rates of 13.5% pre-CT to 7% post-CT and 3.04% pre-CT to 0.73% post-CT, respectively.

Park et al. [10] studied 360 patients undergoing CABG surgery, of which 284 underwent a preoperative CT. Thirty six of the 284 patients scanned (12.7%) displayed severe aortic calcification deemed too high risk for cannulation and clamping which required change in operative strategy. 18 of the 284 patients scanned (6.3%) required changes in bypass conduit selection or grafting strategy. This study showed an improvement in mortality rates from 5.3% no-CT to 1.8% CT.

Furthermore, atherosclerotic disease of the ascending aorta and internal carotid arteries are independent risk factors for postoperative stroke in cardiac surgical patients, and may not be detected by a conventional chest x-ray [1, 12–14]. NCCCT can more accurately determine the extent of aortic calcification to guide cannulation techniques and cross clamping. [3, 4, 7] Additionally, prior to re-sternotomy, NCCCT can delineate the anatomical relationship of mediastinal structures and their proximity to the posterior surface of the sternum, as well as identify previously placed coronary grafts that may be injured upon re-entry [3, 6, 15].

Patients with more atherosclerotic disease in the ascending aorta are significantly more likely to have a higher incidence of carotid artery disease. [12] Pre-operative CD evaluation can be used to identify significant atherosclerosis of the internal carotid artery, but is typically considered for high risk patients that are older than 80, have a carotid bruit, or have a history of cardiovascular disease. [13, 16]

One of the drawbacks to performing NCCCT in all patients scheduled for cardiac surgery is the increased risk of radiation exposure and the cost. [3, 17] The CRICKET study, which began in 2016, aims to evaluate whether the use of ultra-low dose preoperative non-contrast chest CT can reduce stroke rate in cardiac surgery by optimizing surgical strategy and it is doing so with a sample size of 1724 patients. [18] These advancements in CT imaging techniques may reduce unnecessary radiation exposure and will hopefully help favor preoperative CT screening and its utility in improving surgical outcomes. Overall, there are many variables that still need to be investigated regarding the risks and benefits of applying this practice as a universal standard of care.

It is important to highlight that the absence of contrast may have led to limitations in the type of findings found. CT imaging without contrast is typically not ideal for soft tissue findings including malignancy unless significant calcifications are present. Lack of contrast also meant that CT angiography was not performed and vascular characterization was limited. Without contrast and angiography, it is feasible that pathologies such as pulmonary embolisms, metastatic lesions or soft tissue masses may have been missed.

The major limitation of this approach is the initial cost. Nevertheless, routine preoperative NCCCT and CD evaluation in all non-urgent cardiac surgical patients may reduce morbidity and mortality rates in the perioperative and long-term postoperative periods and may lead to decreased healthcare burden and costs overall in the long-run. Since the findings varied and the sample size was small, we were not able to use predictive analytics of patient characteristics in predicting CT findings. Future larger studies should examine whether patients’ characteristics predict CT findings, and explore whether the patient cohorts can be defined when CT evaluation is indicated or not indicated.

## Conclusion

Precise pre-operative evaluation of patients scheduled for heart surgery is extremely important to prevent intra-operative and post-operative complications. Pre-operative NCCCT and CD are typically used only for elderly and/or for high-risk patients. We investigated the use of these pre-operative imaging modalities in all 500 heart surgery patients, regardless of age or risk factors. Based on our results, pre-operative NCCCT and CD were found to be effective at identifying heart, lungs, large vessels, mediastinal, and upper abdomen findings, as well as other incidental findings which may have changed the operative strategies and/or the post-operative treatment and follow-up. In our department, we performed a routine pre-operative evaluation by both NCCCT and CD for all patients scheduled for heart surgery, regardless of their age or risk factors. It is important to emphasize that preforming preoperative NCCCT and CD to all cardiac surgery patients is associated with increased cost, as well as it could pose a risk for younger women at a reproductive age. Therefore, future studies should identify a population that would benefit most from the preoperative screening, based on age, disease etiology and severity, as well as associated risk factors. In addition, more studies should be conducted to evaluate how these studies affect long-term morbidity and mortality.

## Data Availability

Please contact the authors for data requests.

## Abbreviations

NCCCT: Non-contrast chest computerized tomography
CT: Computerized tomography
CD: Carotid arteries Doppler
CABG: Coronary artery bypass graft surgery
